# Isolation, Characterization, Crystal Structure Elucidation, and Anticancer Study of Dimethyl Cardamonin, Isolated from *Syzygium campanulatum* Korth

**DOI:** 10.1155/2014/470179

**Published:** 2014-10-28

**Authors:** Abdul Hakeem Memon, Zhari Ismail, Abdalrahim F. A. Aisha, Fouad Saleih Resq Al-Suede, Mohammad Shahrul Ridzuan Hamil, Suzana Hashim, Mohammed Ali Ahmed Saeed, Madeeha Laghari, Amin Malik Shah Abdul Majid

**Affiliations:** ^1^Department of Pharmaceutical Chemistry, School of Pharmaceutical Sciences, Universiti Sains Malaysia, 11800 Minden, Penang, Malaysia; ^2^EMAN Research and Testing Laboratory, School of Pharmaceutical Sciences, Universiti Sains Malaysia, 11800 Minden, Penang, Malaysia; ^3^Department of Pharmaceutical Technology, School of Pharmaceutical Sciences, Universiti Sains Malaysia, 11800 Minden, Penang, Malaysia

## Abstract

*Syzygium campanulatum* Korth is an equatorial, evergreen, aboriginal shrub of Malaysia. Conventionally it has been used as a stomachic. However, in the currently conducted study dimethyl cardamonin or 2′,4′-dihydroxy-6′-methoxy-3′,5′-dimethylchalcone (DMC) was isolated from *S. campanulatum* Korth, leaf extract. The structural characterization of DMC was carried out by making use of various techniques including UV, IR, NMR spectral followed by LC-MS, and X-ray crystallographic techniques. For determining the purity of compound, highly effective techniques including TLC, HPLC, and melting point were used. The cytotoxicity of DMC and three different extracts of *S. campanulatum* was evaluated against human colon cancer cell line (HT-29) by three different assays. DMC and ethanolic extract revealed potent and dose-dependent cytotoxic activity on the cancer cell line with IC_50_ 12.6 and 90.1 *µ*g/mL, respectively. Quite astonishingly to our knowledge, this is the very first report on *S. campanulatum* as being a rich source (3.5%) of DMC, X-ray crystallography, and anticancer activity on human colon cancer cells.

## 1. Introduction

Chalcone is merely one of the many types of flavonoid phytochemical group. Chalcones are assumed to be precursors of flavones in the biosynthesis of flavonoids. Structurally, chalcones possess 1,3-diaryl-2-propen-1-one moiety in which the two aryl groups are linked together by three carbons (carbonyl and an *α*, *β*-unsaturated system) [1]. Chalcones are yellow-coloured compounds having numerous biological activities including cytotoxic [2], anticancer [3], anti-inflammatory, antioxidant, analgesic, antibacterial, antifungal, and antiprotozoal [4]. Chalcones reserves are inexhaustible in all parts of edible plants.* Syzygium campanulatum* Korth (Myrtaceae) is a shrubby evergreen angiosperm, which is dicotyledonous, tropical or subtropical, and monsoonal, with a height ranging from 2 to 20 m, indigenous to Malaysia and Thailand. It is a commonly found ornamental tree planted along roads parks and public places. On crushing, its leaves produce a fragrance that is like that of cinnamon. It is known by sundry vernacular names such as pokok kelat paya, ubah laut (East Malaysia), Chinese red-wood (Chinese name), red lip, wild cinnamon, Australian brush cherry, and kelat oil [5]. Albeit traditionally being used as stomachic, to date no pharmacognostical and phytochemical profiling is reported on* S. campanulatum* except isolation of betulinic acid [6]. The compound DMC has been isolated from* C. operculatus* [7],* M. serrata* [8], and* S. samarangense* [9]. DMC possessed hepatoprotective [10], cytoprotective [11], anti-inflammatory [12], antifungal, antibacterial [8], anti-TB [13], antiviral [14], antispasmodic [15], antidiarrhoeal [16], antihyperglycaemic [17], anti-MDR [18, 19], and antiapoptotic effect [20]. DMC has been evaluated for its cytotoxic activity on various human cancer cells including liver cancer (SMMC-7721s), HeLa (SPC-A-1, 95-D, and GBC-SD) [7], colon (SW-480) [9], leukemia (K562) [21], colorectal carcinoma (HCT-116 and LOVO) [22], breast (MDA-MB-453), and ovarian [23] cell lines.

In the currently ongoing study, for the first time DMC was isolated from* S. campanulatum* leaf extract at high percentage yield. The compound was characterized using NMR and LC-MS, and its cytotoxic activity was performed on human breast cancer cells. Chemical structure of DMC was further authenticated by X-ray crystallography. Three different extracts of* S. campanulatum* were also subjected to phytochemical profiling versus alkaloids, tannins, the total flavonoids, polyphenols, saponins, polysaccharides, and proteins content. In addition to that, the extracts were also screened for antioxidant and cytotoxic activity* in vitro*. In present work, DMC was evaluated for its potential antiproliferative effect against human colon cancer cell lines. Furthermore, an attempt was made to understand the potency of DMC cytotoxicity; three different assays, that is, cell viability assay, migration, and clonogenicity assays, were performed on selected human colon cancer cells.

## 2. Materials and Methods

### 2.1. Plant Material

The green leaves of* S. campanulatum* Korth were collected in March 2013, from the main campus of University Sains Malaysia, Penang, Malaysia. The plant was authenticated by the Herbarium of School of Biological Sciences, USM, where a voucher specimen was deposited (Ref. number 11047).

### 2.2. Chemicals and Reagents

RPMI 1640 medium, trypsin, and heat inactivated fetal bovine serum (HIFBS) were obtained from Gibco, UK, and 5-fluorouracil 99% HPLC grade, phosphate-buffered saline (PBS), penicillin/streptomycin (PS) solution, 3-(4,5-dimethylthiazol-2-yl)-2-5-diphenyltetrazolium bromide (MTT) reagent, and crystal violet were purchased from Sigma-Aldrich, USA.

### 2.3. Cell Lines and Culture Conditions

Human colon adenocarcinoma cell line (HT-29, ATTC HTB-38) was purchased from ATCC (Rockville, MD, USA). The cells were cultured in RPMI supplemented with 10% HIFBS and 1% PS. Cells were cultured in a 5% CO_2_ in a humidified atmosphere at 37°C.

#### 2.3.1. Extraction

The green leaves were washed under tap water and dried at 45°C in oven for 3 days and ground to fine powder using electric grinder (Retsch, Germany). The powdered leaves (500 g) were separately extracted by various methods including soxhlet with 1 : 1 n-hexane-methanol (extract 1) and ethanol (extract 2), maceration with 1 : 1 ethanol-water (extract 3), and reflux with water (extract 4) for 24 h. The extracts were filtered using Whatman filter paper number 1, and the filtrates were evaporated to dryness at 40°C by using rotary evaporator (Buchi, USA). The crude extracts were kept for 12 h at 45°C in oven to ensure complete dryness. Extract 1 was selected for isolation of DMC, while the remaining three extracts were selected for evaluation of cytotoxicity and phytochemical profile of* S. campanulatum* plant. Stock solutions at 10 mg/mL of extracts 2, 3, and 4 were prepared in 100% dimethyl sulfoxide (DMSO) and further diluted with cell culture medium.

#### 2.3.2. Isolation of DMC

The crude n-hexane-methanol (1 : 1) extract (20 g) of* S. campanulatum* was ground into fine powder using mortar and pestle. Fine powdered crude extract was subjected to flash column chromatography under vacuum using column (10 × 7 cm) packed with silica gel with particle size of 0.063–0.200 mm, 230–400 mesh. The mixture of n-hexane-ethyl acetate (250 mL) by decreasing having hexane ratios 100, 92.5, 60, 30, 15, and 0% was used for obtaining 17 fractions of the extract. Each fraction was collected separately in (500 mL) conical flask and kept in fume hood at room temperature (25°C) for evaporation for 3-4 days. A yellow orange solid (3.8 g) appeared in fractions 7 and 8 and was collected for further investigation.

#### 2.3.3. Crystallization

Solid obtained from fractions 7 and 8 was dissolved separately in n-hexane-ethyl acetate (15 : 1) and allowed to evaporate at room temperature (25°C). Crystals appeared with some impurities which were washed slowly in pure n-hexane. To obtain more pure crystals, solids were dissolved in n-hexane by adding 2 drops of methanol. Solution was kept in fume hood to evaporate and fine yellow orange crystals were obtained. These crystals were redissolved in methanol (2 mL) and allowed to stand 24 h at room temperature (25°C) to obtain fine needle type orange crystals. Purity of crystals was analyzed by performing melting point, HPTLC, HPLC, UV, IR NMR, and X-ray crystallography.

#### 2.3.4. Characterization of DMC

**Figure 1:**
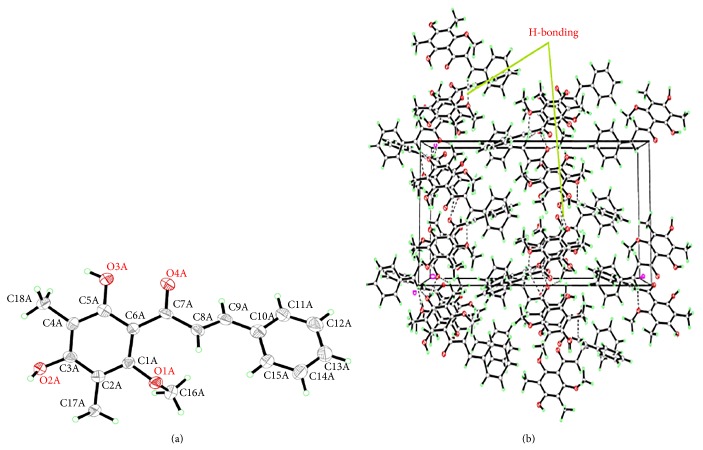
(a) Stereochemical structures of dimethyl cardamonin. (b) Crystal packing of dimethyl cardamonin. The molecules packed in monoclinic crystal system through intermolecular hydrogen bonds shown as dashed lines.

The purified orange yellow crystals of DMC were characterized by FTIR spectrophotometer (Perkin Elmer Spectrum one FTIR spectrometer, USA) using potassium bromide (KBr) disc method. The IR spectrum was scanned at infrared region of 400–4000 cm^−1^. The sample was prepared in HPLC grade methanol (10 mg/mL) and was filtered through 0.45-micron filter. The spectrophotometer was controlled by computer with UV WinLab 25 software. Furthermore, the purified DMC was analyzed by 2D-NMR spectra (FT-NMR spectrometer, Bruker 500 MHz) in* deuterated* methanol (MeOD). The NMR peaks were labelled as singlet (s), doublet (d), triplet (t), and multiplet (m); chemical shifts were referenced with respect to solvent signals. The single crystals obtained were analysed by Bruker SMART APEX2 CCD area detector diffractometer (Figure 1). The molecular graphics were constructed by Mercury 3.1 Development (Build RC5) software.

Mass spectra (*n* = 5) of DMC were determined using a micrOTOF-Q ESI mass spectrometer (Waters) coupled with Agilent HPLC 1200 series, USA. The sample was prepared in MS grade methanol and 2 *μ*L sample was injected directly into the ES source at a flow rate of 5 *μ*L/min. The MS conditions were column; ACQUITY UPLC BEH C18, 1.7 *μ* (Waters, Ireland), mobile phase; solvents A and B: doubled distilled deionised water and acetonitrile, respectively, both with 0.1% formic acid (100 *μ*L/100 mL) at a flow rate 0.3 mL/min positive ion mode; gas (N_2_); temperature, 350°C; flow rate, 10 L/min; nebulizer pressure, 15 psi; sample infusion flow rate 20 *μ*L/min; HV voltage, 4.0 kV; octopole RF amplitude, 150 Vpp; skim 1 voltage, −38.8 V; skim 2 voltage, −6.0 V; cap (KV) 3 V; sampling cone 40, extraction cone 4, and scan range,* m/z* 100–1000 units. Acquired mass spectra represented the average of *n* = 5 spectra.

#### 2.3.5. Quantification of DMC in Different Extracts of* S. campanulatum*

HPLC analysis was performed using Agilent HPLC 1260 system, on ZORBAX Eclipse Plus Phenyl-Hexyl column (4.6 × 250 mm, 5 microns). The mobile phase consisted of A (acetonitrile) and B (0.1% H_3_PO_4_ in water). The elution program was isocratic at 60% (A) and 40% (B) for 20 min, at 1 mL/min flow rate. Sample injection volume was 10 *μ*L (500 ppm) methanolic DMC solution at *λ*_max⁡_ 330 nm. The purity of DMC was assured from chromatogram and plant extracts were analyzed for the presence of DMC and the results were calculated as % w/w.

#### 2.3.6. Phytochemical Analysis

Preliminary tests for alkaloids, tannins, flavonoids, glycosides, phenols, steroids, and terpenoids on all three extracts of* S. campanulatum* were performed as described in [24–26]. The above phytochemical groups were found in all three extracts except water extract in which flavonoids, steroids, and terpenoids were not present.

#### 2.3.7. Estimation of Total Phenolics, Flavonoids, Saponins, Polysaccharides, Proteins, and Antioxidant Effect of Three Different Extracts of* S. campanulatum*

Total phenolic contents (TPC) were estimated using a colorimetric assay [27]. Twenty microliters of extracts and gallic acid (GA) in methanol (5 mg/mL) were added to 1.58 mL distilled water followed by 100 *μ*L of Folin-Ciocalteau reagent and incubated for 8 min in the dark at RT. Subsequently, 300 *μ*L of sodium bicarbonate (20%) was added and incubated in the dark at 30°C for 2 h, and absorbance was measured at 765 nm. GA was used as a standard (0.03–5 mg/mL), and the results are expressed in Table 1 as % w/w GA equivalents using GA calibration equation (*y* = 0.000*x* + 0.074, *R* ^2^ = 0.9997).

Total flavonoids content (TFC) was determined using quercetin as a standard [28]. The standard and extracts (500 *μ*L of 5 mg/mL) were added to 0.1 mL 10% (w/v) of aluminium chloride, 0.1 mL of 1 M potassium acetate, 1.5 mL of methanol, and 2.8 mL of water. The reaction mixture was incubated for 30 min at RT, and absorbance was taken at 415 nm. All samples were prepared in triplicate. In blank preparation distilled deionised water was used as constituent of 10% aluminium chloride. Total concentration of TFC was calculated as mg of quercetin (0.6–156 mg/mL) equivalent by using quercetin calibration curve (*y* = 0.008*x* + 0.004,  *R* ^2^ = 0.999).

DPPH scavenging activity was performed as described in [24]. DPPH (0.75 mL) at a final concentration of 0.005% w/v was added to (0.25 mL) three different plant extracts of 1 × 10^3^ to 1 × 10^−6^ *μ*g/mL. The mixtures were incubated in the dark at 30°C for 30 min. Subsequently, absorbance was measured at 517 nm, and DPPH scavenging effect was calculated as follows.

DPPH scavenging effect = (1 − (absorbance of samples-blank)/(absorbance of negative control-blank)) × 100 [6]. The results are presented as mean percentage inhibition ± SD (*n* = 3).

Total glycosaponins (TGS), total polysaccharides (TPS), and total proteins were estimated from water, ethanol, and ethanol 50% extracts of* S. campanulatum* using methods as described in [29]. Results are summarized in Table 1.

#### 2.3.8. TLC Analysis of DMC in* S. campanulatum* Extracts

TLC technique was performed to check the purity of isolated DMC and presence of DMC in* S. campanulatum *extracts. TLC plates coated with 0.25 mm silica gel 60 F254 (Merck, Germany) were used. The methanolic solution 10 *μ*L of 1 mg/mL DMC and three plant extracts were applied on plate using microsyringe. Plate was developed in saturated chamber using n-hexane-ethyl acetate-formic acid (7 : 3 : 0.1). The plate was dried at 110°C for 30 min and examined at 256 and 350 nm wavelengths.

#### 2.3.9. Colour Reactions of DMC

DMC possesses carbonyl group (C=O) of the chromogen (Ar–COCH=CH–Ar) as an intermediate which can produce deeper colour during the reaction by a phenomenon known as “Halochromy” [30]. The chemicals used for colour tests of DMC are FeCl_3_ (alcoholic), H_2_SO_4_ concentrated, H_2_SO_4_-HNO_3_ (mixture), H_2_SO_4_-(CH_3_COO)_2_O (mixture), NaBH_4_-HCl, SbCl_5_, and H_3_BO_3_-C_6_H_8_O_7_ or Wilson's boric test.

*FeCl*_*3*_ *(Alcoholic) Colour Complex with DMC.* DMC (15 mg) was dissolved in ethanol (1 mL) followed by two drops of freshly prepared alcoholic FeCl_3_ solution. Instantaneously definite shades of colours (i.e., blue, wine red, blue black, violet, or green colours) were produced.

*H*_*2*_ *SO*_*4*_ *(Concentrated) Colour Complex with DMC.* DMC produced transient intense red colour carbonium ion complex when dissolved in H_2_SO_4_ (concentrated). DMC exhibits a decent halochromic effect when wetted with concentrated H_2_SO_4_. The resonance must be considered as extended to the benzene ring.

*H*_*2*_ *SO*_*4*_ *-HNO*_*3*_ *(Mixture) Colour Complex with DMC.* On the addition of concentrated HNO_3_ to solution of DMC in H_2_SO_4_ (concentrated) no any little characteristic change in colour was observed. This is due to nitration of chalcone on carbon 3′ rather than oxidation and the resulting nitrochalcone. In this test DMC has (–CH_3_) group on 3′-C so nitronium ion complex with DMC is not possible.

**Figure 2:**
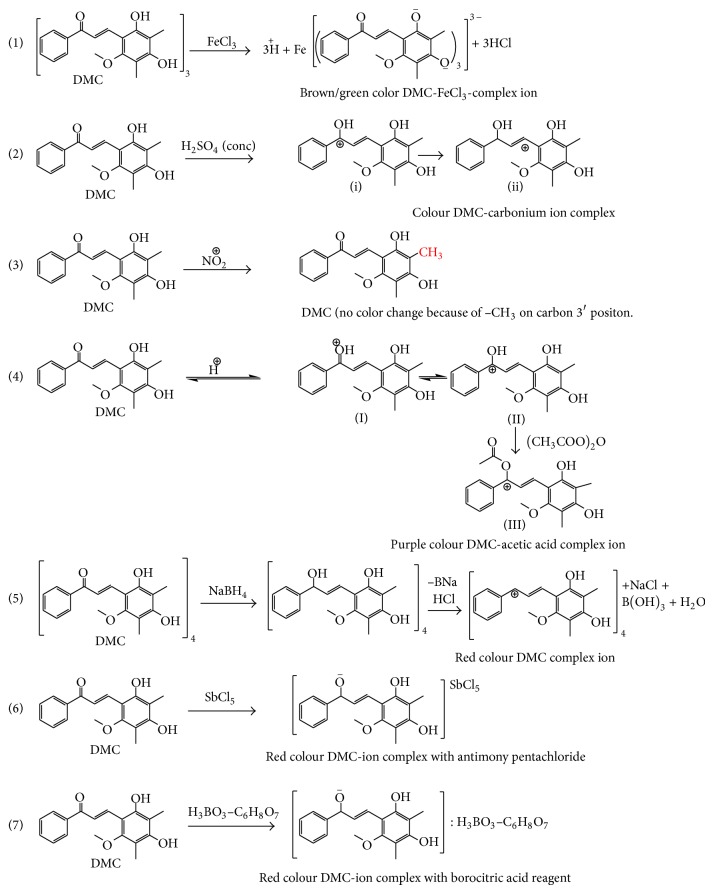
Schematic diagram of colour reactions of dimethyl cardamonin with different chemical reagents.

*H*_*2*_ *SO*_*4*_ *-(CH*_*3*_ *COO)*_*2*_ *O (Mixture) Colour Complex with DMC.* Substituted (–OCH_3_ and –OH) chalcones in CH_3_COOH solution yield deep colour (orange to purple) when treated with a drop or two of H_2_SO_4_ (concentrated). Two drops of H_2_SO_4_ (concentrated) added to (0.2% w/v) acetic anhydride solution of DMC (5 mg) at 10–15°C. DMC changes from orange to purple colour. Due to the addition of acetic anhydride to the DMC in H_2_SO_4_ (concentrated), bathochromic shift has been reorganized in terms of stability conferred on the carbonium ion (II → III) by acetylation with acetic anhydride (Figure 2).

*NaBH*_*4*_ *-HCl (Mixture) Color Complex with DMC.* Na (or K) BH_4_ is a selective reagent for the reduction of carbonyl (–CHO and C=O) group to the corresponding alcohol. DMC with NaBH_4_-HCl (mixture) produced instantly red colour. In a 25 mL Erlenmeyer flask, NaBH_4_ (33 mg) was added in 1 mL of methanolic DMC (133 mg) in small portions with swirling that the temperature did not exceed 45°C. The reaction is exothermic, so NaBH_4_ (33 mg) should be added slowly. After addition of NaBH_4_ (33 mg), mixture was boiled for 2 min. The mixture was allowed to cool and red colour DMC ion complex was produced on addition of ethanolic-HCl mixture (Figure 2).

*SbCl*_*5*_ *Colour Complex with DMC.* DMC treated with SbCl_5_ in CCl_4_ produced intense red or violet precipitates, which are characteristically different from the yellow or orange precipitates produced by flavonols, flavones, and flavanones. DMC (5 mg) dissolved in 5 mL of anhydrous CCl_4_ and 1 mL (2% anhydrous CCl_4_) solution of SbCl_5_ was added.

*H*_*3*_ *BO*_*3*_ *-C*_*6*_ *H*_*8*_ *O*_*7*_ *(Wilson's Boric Test) Colour Complex with DMC.* Chalcones with an* ortho*-(–OCH_3_ or –OH) group give positive colour reaction with borocitric acid reagent [31]. Two separate solutions were prepared in absolute acetone: (a) boric acid (saturated solution) and (b) dry citric acid (0.5 gm) in absolute acetone (5 mL). Citric acid was allowed in air (30–40°C) for complete efflorescence and then heated in a thin layer for 2 h at 100°C. DMC (5 mg) was dissolved in 1 mL of dry acetone and then divided into two equal portions. In the first portion 2 mL of boric acid-citric acid-acetone reagent (solutions C and D) and in the second portion 0.5 mL of solution D were added. After few minutes the colours of both portions were compared and stronger red colour in the first portion containing boric acid-citric acid reagent was regarded as a positive reaction for 5-(–OH or –OCH_3_) chalcone (DMC).

#### 2.3.10. Cell Viability Assay

Viability of the HT-29 cells was determined by the MTT test as described by [32, 33]. Cells (70–80% confluency) were treated with various concentrations of extract and DMC. Similarly, 5-fluorouracil with serial concentration is used as standard reference drug. After 48 h incubation, 20 *μ*L of MTT solution (5 mg/mL in PBS) was added and incubated for an additional 3-4 h. Subsequently, the medium was aspirated carefully, and 150 *μ*L of dimethyl sulfoxide (DMSO) was added. After incubation for 15 min, the optical density was measured at 570 nm using a high-end Tecan M200 Pro multimode microplate reader. Data were recorded and analyzed for the assessment of the effects of the test substance on cell viability and growth inhibition. The IC_50_ values were calculated using regression equation as explained before [34]. The results are presented as the average percentage viability to the negative control (1% DMSO). The percentage of cell viability was calculated using the following formula: % cell viability = (absorbance of treated/absorbance of untreated) × 100.

The percentage of inhibition was plotted against the concentration in Microsoft excel and the IC_50_ was calculated using the regression equation.

#### 2.3.11. Migration Assay of HT-29 Cells

The effect of DMC on the migration of HT-29 cells was examined by the wound healing assay. HT-29 cells were maintained in 6-well plate in RPMI until 100% confluent monolayer growth was obtained. As previously described [35] the monolayer was scratched with a sterile 200 *μ*L micropipette tip and then washed with PBS to remove the unattached cells and to smoothen the edges of the scratch. After that, 2 mL of RPMI was added to each well followed by addition of DMC at 10 and 20 *μ*g/mL final concentrations. 5-Fluorouracil (20 *μ*g/mL) and vehicle (1% DMSO) were added as positive and negative controls, respectively. Subsequently, 6–8 microscopic fields per well were photographed at 0, 12, and 24 h using an AMG EVOS fI digital microscope. The width of the cell-free area was measured using Leica Qwin software.

The percentage of wound closure was then calculated relative to zero time using the following formula:

 % wound closure = (1 − (the width at the indicated times (h)/the width at zero time)) × 100%. The results are displayed as average ± SD (*n* = 6).

#### 2.3.12. Clonogenicity Assay

The effect of DMC on HT-29 colony formation was evaluated as described by [36]. HT-29 cells in log phase growth were prepared in a single-cell suspension before being placed in a 6-well plate (500 cells/well). The cells were incubated for 24 h to facilitate their attachment and treated with various concentrations of ethanol extract, DMC, 5-fluorouracil (3 *μ*g/mL) as a positive control and 1% DMSO as a negative control. Following additional 48 h incubation, treatments were removed, and cells were incubated in a drug-free medium for an additional 10 days until large colonies were produced. Colonies were then fixed with 4% paraformaldehyde for 30 min and stained with 0.2% (w/v) crystal violet for 20 min. The colonies with >50 cells were counted under a stereomicroscope and the plating efficiency (PE) and survival fraction (SF) were calculated. Results are expressed as the mean ± S.D (*n* = 3).

## 3. Results and Discussion 

### 3.1. Extraction and Isolation of the Active Compound

Dried green leaves of* S. campanulatum* were extracted using soxhlet with n-hexane-methanol (1 : 1) and crude extract was obtained. The crude extract was subjected to flash column chromatography using increasing concentration of ethyl acetate in n-hexane starting with 100% n-hexane. Seventeen fractions were obtained; yellow solid mass of DMC appeared in fractions 7 and 8 (n-hexane-ethyl acetate 85 : 15) and was recrystallized using a mixture of methanol-n-hexane (1 : 9).

### 3.2. Spectroscopy

DMC was obtained as orange yellowish needle type crystals, having MP: 126°C. The UV-Vis spectra of DMC showed absorption at *λ*_max⁡_ 332 nm indicating its chalcone characteristics [37]. FTIR spectrum showed a strong and sharp vibrational band at 3401 cm^−1^ that indicated the presence of (–OH) group, 2932 cm^−1^ (CH_2_) [38], 1623 cm^−1^ (C=O), and 1545 cm^−1^ (CH=CH) [39]. The presence of alkyl groups was assigned by 2850 and 2939 cm^−1^ two vibrational bands [40]. These prominent characteristic functional groups indicated the presence of chalcone, a class of compounds based on* trans*-1,3-diaryl-2-propen-1-ones backbone [41, 42]; DMC was also characterized by 1H and 13C NMR. The 13C DEPT-135 and 145 NMR spectra recorded in MeOD at 125.75 MHz at 25°C are shown in Figures S1, S2-A, and S2-B, respectively (see the Supplementary Material available online at http://dx.doi.org/10.1155/2014/470179). The 2D-HSQC and HMBC NMR spectra of DMC are shown in Figures S3 and S4, respectively. Supplementary Figures S5 and S6 illustrate the characteristic diagonal component and cross peaks of DMC found in 2D-TOCSY and 2D-COSY NMR spectra, respectively. The NMR data of these spectra were also matched with previous reported NMR spectra [43, 44]. The molecular weight of DMC was determined by liquid chromatography-mass spectroscopy (LC-MS); one fragmented molecular ion peak was observed at 195 because of cleavage of molecule at *α*-unsaturated carbon as the main fragmentation and one peak at 299 as the main compound shown in supplementary Figures S7-A and B.

### 3.3. Crystallography

DMC single crystal was found appropriate for X-ray crystallographic study. The crystals appeared as orange yellow needle type structure. A perspective view of the crystal structure is illustrated in Figure 1. DMC intramolecular geometry was analyzed using monoclinic space group P21 (number 4), a knowledge base molecular geometry obtained from Cambridge Structural Database. Each unit of DMC consists of two benzene rings, one carbonyl carbon and *α*-*β*-unsaturated carbons. In addition two hydroxyl, methyl, and one methoxy groups are attached on one benzene ring. Crystal data and structure refinement details of DMC are as follows: formula: C18 H18 O4, formula weight: 298.32, crystal system: monoclinic, space group: P21 (number 4), a: 10.8917 (4), b: 24.6366 (10), and c: 11.4428 (5) (Å), respectively, and alpha: 90°, beta: 99.387° (2), gamma 90°, V: (Å^3^) 3029.4 (2), Z: 8, D (calc): 1.308 g/cm^3^, abs. coeff.: Mu (CuKa) 0.752/mm, F (000): 1264, crystal size: 0.09 × 0.14 × 0.52 mm, temperature: 100 K, radiation: Cu Ka 1.54178 Å, *θ* min., max. 3.9, 69.3°, dataset: −13 : 13; −29 : 29; −13 : 13, tot., uniq. data: 28817, R (int): 0.045, observed data [*I* > 2.0 sigma (*I*)]: 9904, 8774, Nref, Npar: 9904, 806, and R, wR2, S: 0.0851, 0.2586, 1.03. The selected bond lengths and angles of DMC crystallographically are shown in Figure 1 and are listed in supplementary data 1. The crystal packing (Figure 1) shows that the DMC molecules are connected in such a way they form a crisscross chain by C–H⋯*π*(arene)⋯H bonds along the *c* axis. There are also intermolecular hydrogen bonding interactions in which C-11 (donor for H-bond), via H-11–O in neighboring molecules at *x*, 1 − *y*, 1 − *z*, to produce a three-dimensional network [45]. In chalcones dihedral angle between the two benzene rings is 13.0(1)° and the dihedral angle from the plane of C7–C9 to the benzene rings (C1–C6) is 13.8(1)° and (C10–C15) is 2.6(1)°. This indicates that the central C7–C9 portion lies nearly in the benzene ring plane of C10–C15 but is quite displaced out of the other benzene ring of C1–C6 [42].

### 3.4. Chromatography

The purity of DMC found to be 98% using HPLC by injecting 10 *μ*L (500 ppm) methanolic DMC solution was checked by performing HPLC analysis using isocratic water-acetonitrile (4 : 6) solvent system. DMC was detected at a *λ*_max⁡_ of 330 nm with a retention time of 9.9 min and 98% purity (Supplementary Figure S8).

### 3.5. Chemical Reactions

Characterization of different functional groups, that is, –OH, –CH_3_, –OCH_3_, >C=O, and CH=CH, present in DMC molecule was also confirmed by performing various colour reactions for chalcones. Figure 2 indicates that the DMC forms colour complex ions (CCI) and is characterised by strong halochromism, due to the fact that the (>C=O) group activated by the (–CH=CH–) declares the presence of the (–COCH=CH–) group linked with two phenyl rings.

Different colour complex ions (CCI) of DMC indicated that alc. FeCl_3_ produce CCI only with (–OH) chalcones, while NaBH_4_-HCl and Ac_2_O-H_2_SO_4_ reagents indicate that the chromogen involved in formation CCI of chalcone is (Ar–CO–CH=CH–Ar). This reaction involves the attack by (–C^+^=O) at the carbonyl carbon of the chalcones chromogen producing (–C^+^–(OAc)–CH=CH–) CCI [31]. The H_2_SO_4_-concentrated HNO_3_ reagent did not produce CCI because in DMC molecule 3′-C is not free and occupied by (–CH_3_) group.

### 3.6. Inhibition of HT-29 Proliferation by Ethanolic Extract of* S. campanulatum* and Isolated DMC

**Figure 3:**
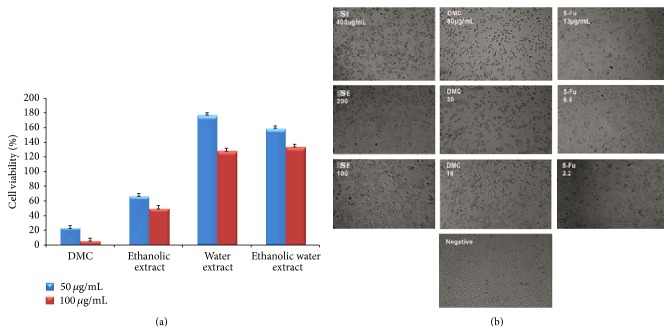
(a) Cell viability (%) ± SD (*n* = 3) tested on DMC and three different extracts of* S. campanulatum *at two selected concentrations. (b) Dose-dependent antiproliferative effect of* S. campanulatum *ethanolic extract and DMC on HT-29 cell lines was assessed by MTT assay.

The MTT cell proliferation assay was executed with great care in order to study the effect of three different extracts and DMC isolated compound from the leaves of* S. campanulatum* on HT-29 cell line. The screening on ethanolic, water, and 50% ethanol extracts of* S. campanulatum* and DMC compound isolated from* S. campanulatum* leaves at two selected concentrations that were 50 *μ*g/mL and 100 *μ*g/mL confirmed that ethanolic extract and DMC have strong cytotoxic effect against HT-29 cells as compared to water and 50% ethanolic extract (Figure 3(a)). In correspondence to Figure 3(b), both ethanolic extract of* S. campanulatum *and DMC inhibited the proliferation on HT-29 in dose-dependent manner. The median inhibitory concentrations (IC_50s_) for ethanolic extract of* S. campanulatum* and DMC were then calculated authentically and with the accurate precision from the dose response curves and were found to be 90 and 12.6 *μ*g/mL, respectively. For further advancement and accuracy, 5-fluorouracil, a standard therapeutic drug for cancer, was used as a positive control, and it showed significant toxicity towards HT-29 cells with an IC_50_ of 27.6 *μ*g/mL. In comparison to previous study in which hexane and methanolic extracts of* S. campanulatum* were reported active against HCT-116, HT-29, MCF-7, T47D, and rat hepatoma cells using cell-enzyme based* in vitro* assay [46] unlikely in present study ethanolic extract of* S. campanulatum* and pure DMC are studied for the first time against HT-29 cell line using three different assays.

### 3.7. DMC Inhibited Migration of HT-29 Cells

**Figure 4:**
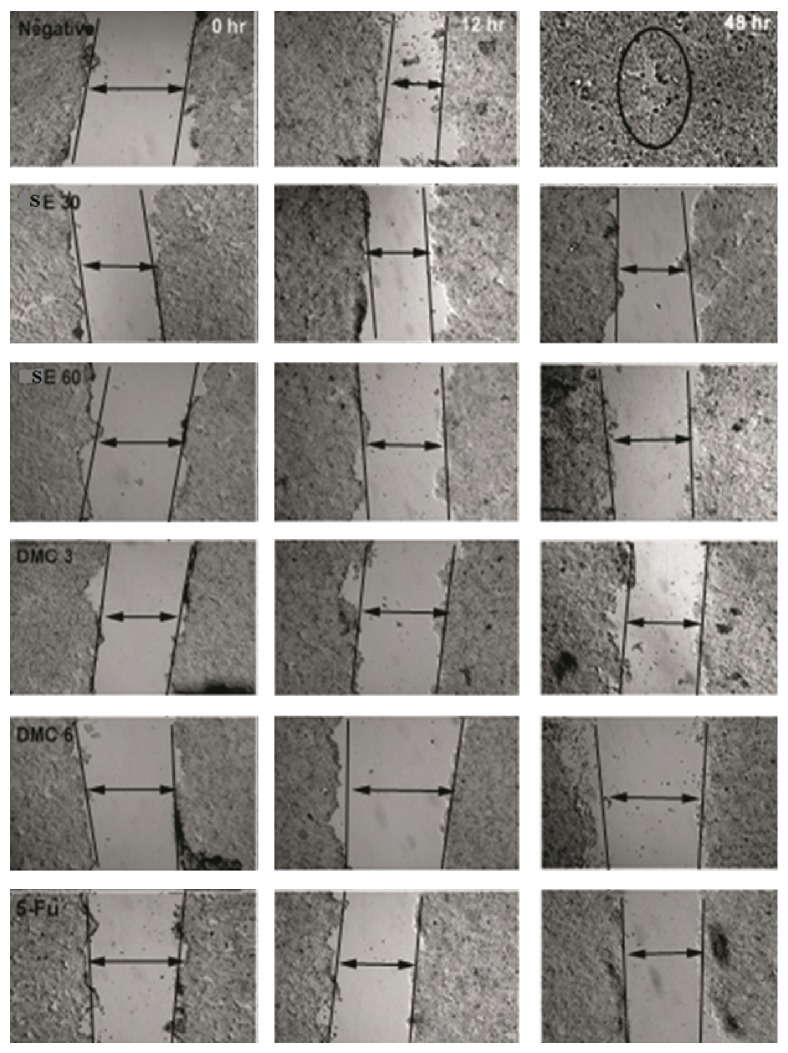
Migration assay of HT-29 cells treated with different concentrations of DMC and ethanolic extract of* S. campanulatum *at 0, 12, and 48 h.

**Figure 5:**
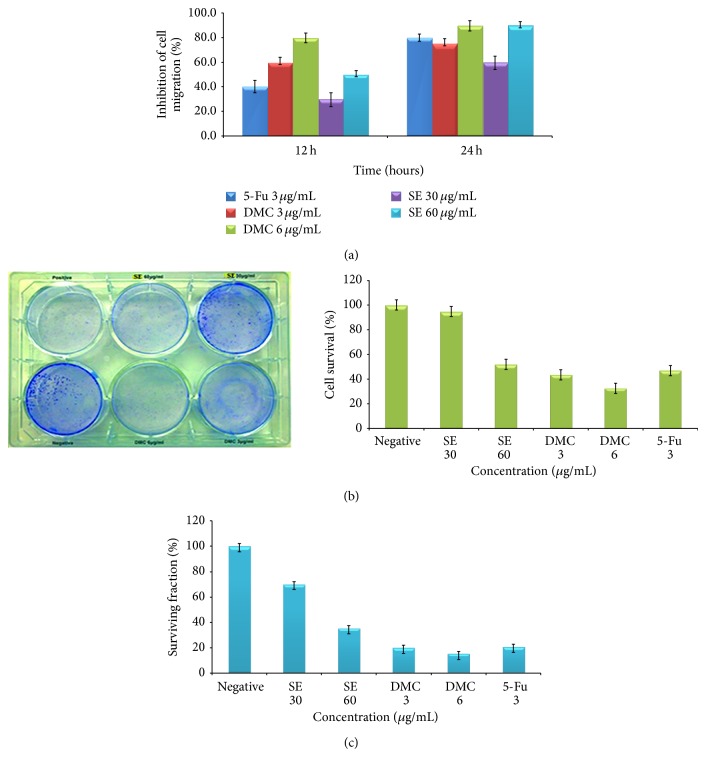
(a) The inhibitory effects of DMC and ethanolic extract of* S. campanulatum* on the migration property of HT-29 cells, which is a characteristic of metastasis of the cancer cells. (b) Clonogenic cell survival (%) of HT-29 treated with 1% DMSO as a negative control, 3 *μ*g/mL of 5-fluorouracil as positive control, and indicated concentrations of* S. campanulatum* ethanolic extract and DMC. (c) The percentage of surviving fraction obtained after the treatment with DMC and ethanolic extract of* S. campanulatum*. The percentage of surviving fraction of the HT-29 colonies was decreased with increasing concentration of DMC and ethanolic extract* S. campanulatum*, mean ± SD (*n* = either 6 or 10).

Wound healing assay was used to examine the effects of DMC and ethanolic extract of* S. campanulatum *on the migration of HT-29 cells. For improved results, this was executed on the experimental lines: the percentage of inhibition of wound closure in the presence and absence of DMC was calculated after every 12 and 48 h, relative to the zero time. The results of the percentage of inhibition of wound healing assay are as depicted in Figure 4. The results sharply outlined the fact that the percentage of wound closure after 48 h was almost 100% in the untreated cells, whereas, in treated group, the wound remained open even after 48 h period time. The percentage of inhibition of migration of HT-29 cancer cells is shown in Figure 5(a). DMC (3 *μ*g/mL), at every 12 and 48 h, inhibited the migration of HT-29 cells up to 60 and 75%, while 6 *μ*g/mL concentration of DMC inhibited cell migration at 80 and 90% at the same duration of 12 and 48 h, respectively. The ethanolic extract of* S. campanulatum* after 12 and 48 h at 30 and 60 *μ*g/mL inhibited the HT-29 migration 30–60 and 50–90%, respectively. The positive control 5-fluorouracil (3 *μ*g/mL) showed 40 and 80% inhibition in migration of HT-29 cells after 12 and 48 h, respectively.

### 3.8. DMC Obstructed HT-29 Colony Formation

Treatment of HT-29 cells with DMC in the concentration range of 3–6 *μ*g/mL caused dose-dependent inhibition of the HT-29 clonogenicity. The cell survival (%) graph for DMC is depicted in Figure 5(b). At concentrations of 3 *μ*g/mL, DMC inhibited the colony formation of HT-29. At 6 *μ*g/mL concentrations, DMC significantly inhibited the formation of colonies; the survival fraction (SF) at 6 and 3 *μ*g/mL of DMC was 15 ± 4.8% and 21 ± 2.6% and 25 ± 4.4, 70 ± 4.0 after ethanol extract treatment at the concentration of 60 *μ*g/mL and 30 *μ*g/mL, respectively. At high concentration, ethanolic extract showed cytotoxic effect while at low concentration it was cytostatic as evident by decreased survival fraction (SF). These results can be compared with the standard reference, 5-fluorouracil, at 3 *μ*g/mL that showed 20% survival fraction (Figure 5(c)).

In the last 10 years about ninety chalcones with antitumor activities were reported [1] through induction of apoptosis, cell signal transduction, redifferentiation, and induction of apoptosis under hypoxia [1, 21, 22, 47]. However, in the present study the cell migration study was conducted to represent important step in metastasis of cancer cells. The result is shown as percentage of inhibition of migrating cells relative to negative control. Significant reduction in HT-29 cell mobility was achieved 100% after 12 h at 6 *μ*g/mL for DMC and for ethanolic extract of* S. campanulatum* after 48 h at 60 *μ*g/mL of treatment 98.4% relative to negative control.

## 4. Conclusions

In the present study, the isolation of DMC from dried leaves of* S. campanulatum *with its purification and detailed chemical structural characterization and anticancer activity is reported. It has been observed that dimethyl cardamonin (DMC) and ethanolic extract of* S. campanulatum *possess strong cytotoxic activity against human colon cancer cells. Further* in vivo* experiments will then be conducted to evaluate the potential of DMC and ethanolic extract of* S. campanulatum* for the treatment of colon cancer in animals in order to include DMC and ethanolic extract in the clinical trials.

## Supplementary Material

Figure-S: shows ^1^H-NMR-Spectra of DMC, S1:^13^C-DEPTQ-135-NMR-Spectra of DMC. S2: ^13^C-DEPTQ-145-NMR-Spectra of DMC. S3: ^13^C-DEPTQ-all-NMR-Spectra of DMC. S4: HMBC-NMR-Spectra of DMC. S5: 2DCOSY-NMR-Spectra of DMC. S6: 2D-TCOSY-NMR-Spectra of DMC. S7A-B: LCMS fragmentation and Mass analysis of DMC. S8: UV-spectra and HPLC chromatogram. Tables: Supplementary data for crystallography data of DMC.

## Figures and Tables

**Table 1:** Total phenols, glycosaponins, polysaccharides, proteins, and antioxidant values of different extracts of *S. campanulatum*.

S. number	Extract	Phenols (% ± SD)	Glycosaponins (% ± SD)	Polysaccharides (% ± SD)	Proteins (% ± SD)	Flavonoids (% ± SD)	IC_50_ DPPH (*µ*g/mL ± SD)
1	Water	31.2 ± 0.9	87 ± 0.08	1.65 ± 0.2	25.3 ± 0.02	44.9 ± 0.81	240 ± 1.0
2	Ethanolic	31.4 ± 0.36	1.6 ± 0.38	0.68 ± 0.03	30.5 ± 0.04	68.8 ± 0.11	12.8 ± 0.71
3	Ethanolic 50%	39.2 ± 0.37	52.7 ± 0.18	1.04 ± 0.13	25.2 ± 0.01	36.6 ± 0.22	9.51 ± 0.97
