# Supplementary material for: Isolation, Characterization, Crystal Structure Elucidation, and Anticancer Study of Dimethyl Cardamonin, Isolated from Syzygium campanulatum Korth
Source: Evid Based Complement Alternat Med. 2014 Oct 28;2014:470179. doi: 10.1155/2014/470179 (PMC4228816; doi:10.1155/2014/470179)
Supplement: Supplementary file 1 — Figure-S: shows 1H-NMR-Spectra of DMC, S1:13C-DEPTQ-135-NMR-Spectra of DMC. S2: 13C-DEPTQ-145-NMR-Spectra of DMC. S3: 13C-DEPTQ-all-NMR-Spectra of DMC. S4: HMBC-NMR-Spectra of DMC. S5: 2DCOSY-NMR-Spectra of DMC. S6: 2D-TCOSY-NMR-Spectra of DMC. S7A-B: LCMS fragmentation and Mass analysis of DMC. S8: UV-spectra and HPLC chromatogram. Tables: Supplementary data for crystallography data of DMC. [file 470179.f1.docx]

Figure S7

Figure S8
